# Evaluation of Vinca Rosea’s Protective Effects on Hepatic Function in Streptozotocin-Induced Diabetic Wistar Albino Rats

**DOI:** 10.7759/cureus.74166

**Published:** 2024-11-21

**Authors:** Balida Mallikarjuna Rao, T. Vedavijaya, Y. Roja Ramani, Bimalendu Chowdhury

**Affiliations:** 1 Pharmacology and Therapeutics, Meenakshi Academy of Higher Education and Research, Chennai, IND; 2 Pharmacology, Meenakshi Ammal Dental College and Hospital, Chennai, IND; 3 Clinical Pharmacology, Orissa University of Health Sciences, Bhubaneswar, Bhubaneswar, IND; 4 Pharmacology, Roland Institute of Pharmaceutical Sciences, Berhampur, IND

**Keywords:** alt, ast, diabetes, hepatic function, liver enzymes, serum bilirubin, stz, vinca rosea, wistar rats

## Abstract

Background

Diabetes mellitus, characterized by chronic hyperglycemia, often leads to severe hepatic dysfunction, including increased liver enzyme levels and histopathological changes in the liver. Streptozotocin (STZ)-induced diabetic rat models provide a valuable method for evaluating potential therapeutic agents that target hepatic complications. *Vinca rosea*, a medicinal plant with known anti-diabetic properties, has been used traditionally for its hepatoprotective effects, although scientific evidence is limited.

Objective

This study aimed to evaluate the protective effects of *Vinca rosea* leaf extract on hepatic function, including biochemical and histopathological changes, in STZ-induced diabetic Wistar albino rats.

Methods

Thirty male Wistar albino rats were randomly divided into five groups: Normal control (NC), diabetic control (DC), low-dose *Vinca rosea* (LD, 200 mg/kg), high-dose *Vinca rosea* (HD, 400 mg/kg), and positive control (PC, metformin 100 mg/kg). Diabetes was induced by a single intraperitoneal injection of STZ (100 mg/kg). *Vinca rosea* treatment was administered daily for 30 days. Blood samples were collected at 15 and 30 days to assess blood glucose and liver function, including serum bilirubin, aspartate aminotransferase (AST), and alanine aminotransferase (ALT) levels. Liver tissue was collected for histopathological examination. Data were analyzed using analysis of variance (ANOVA), with p < 0.05 considered statistically significant.

Results

The DC group showed significantly elevated blood glucose levels (256 ± 19.8 mg/dL at 15 days and 308.5 ± 13.1 mg/dL at 30 days). *Vinca rosea* treatment significantly reduced blood glucose in a dose-dependent manner, with the HD group showing the greatest improvement (143 ± 12.7 mg/dL at 15 days and 158.5 ± 10.7 mg/dL at 30 days). Liver function markers, including total and direct bilirubin, AST, and ALT, were significantly elevated in the DC group, indicating hepatic damage. *Vinca rosea*-treated groups showed significant improvements in all liver function parameters, with the HD group displaying the most substantial reductions in bilirubin, AST, and ALT levels. Histopathological analysis revealed marked hepatocellular damage in the DC group, including necrosis and ballooning degeneration. In contrast, *Vinca rosea*-treated groups, particularly the HD group, exhibited near-normal liver architecture with minimal damage.

Conclusion

*Vinca rosea* demonstrated significant hepatoprotective effects in STZ-induced diabetic rats by reducing blood glucose levels and improving liver function. These results suggest that *Vinca rosea* could be a promising therapeutic agent for managing diabetes-related hepatic dysfunction.

## Introduction

Diabetes mellitus (DM) is a chronic metabolic disorder characterized by persistent hyperglycemia, arising from defects in insulin secretion, insulin action, or a combination of both [1]. It has emerged as a significant global health issue, with its prevalence increasing at an alarming rate, posing a major burden on healthcare systems and contributing substantially to morbidity and mortality worldwide [2]. The complications associated with diabetes are multifaceted, affecting various organ systems and leading to long-term damage if left uncontrolled. Among these complications, hepatic dysfunction, often referred to as diabetic hepatopathy, has gained attention as an important yet underrecognized consequence of diabetes. This condition encompasses a spectrum of liver abnormalities, including non-alcoholic fatty liver disease (NAFLD), elevated liver enzyme levels, and hepatocellular damage, which can progress to more severe forms of liver injury if not adequately addressed. The pathophysiology of diabetic hepatopathy is complex and involves the interplay of insulin resistance, chronic inflammation, and oxidative stress, which collectively contribute to hepatic steatosis, fibrosis, and potential liver failure if unmanaged [3,4].

Streptozotocin (STZ)-induced diabetes in animal models is a widely recognized and reliable method for studying the pathophysiology of DM, particularly type 1 diabetes. STZ, a glucosamine-nitrosourea compound, is known for its selective toxicity toward pancreatic beta cells due to its uptake via the glucose transporter 2 (GLUT2) receptors, which are predominantly expressed on beta cells. Once inside the cells, STZ induces DNA alkylation, oxidative stress, and mitochondrial dysfunction, leading to beta-cell apoptosis. This destruction of insulin-producing beta cells results in persistent hyperglycemia, which closely mimics the insulin deficiency and metabolic disturbances characteristic of type 1 diabetes [1,2]. The STZ-induced diabetic model not only replicates the hallmark feature of sustained hyperglycemia but also mirrors the progression of complications associated with chronic diabetes. These include nephropathy, neuropathy, retinopathy, and hepatic dysfunction, making it a valuable tool for investigating the systemic effects of diabetes. In particular, liver damage observed in STZ-induced diabetic animals resembles the hepatic manifestations seen in human diabetes. Hyperglycemia-induced oxidative stress, lipid peroxidation, and inflammation play a central role in the development of diabetic hepatopathy, including conditions such as NAFLD and hepatocellular injury [3,4]. The relevance of the STZ model to type 1 diabetes pathophysiology is further underscored by its ability to replicate key features of the disease, including rapid onset of hyperglycemia, insulin deficiency, and subsequent metabolic derangements. This model is also instrumental in studying the progression of diabetes-related complications and evaluating the therapeutic potential of novel interventions aimed at mitigating oxidative stress, inflammation, and organ-specific damage. Furthermore, the reproducibility and simplicity of STZ administration enhance its utility in preclinical research, enabling a deeper understanding of the molecular and cellular mechanisms underlying type 1 diabetes and its complications [5,6]. This model is valuable for evaluating the efficacy of potential therapeutic agents in managing diabetes-related hepatic dysfunction [7,8]. Liver damage in diabetic conditions is commonly evidenced by elevated levels of serum bilirubin, aspartate aminotransferase (AST), and alanine aminotransferase (ALT), which serve as critical biomarkers of hepatic function [9,10]. The underlying cause of liver damage in diabetes is multifactorial and involves several interconnected mechanisms. Chronic hyperglycemia leads to excessive production of reactive oxygen species (ROS), resulting in oxidative stress, which is a key driver of hepatocellular injury. Additionally, insulin resistance, a hallmark of diabetes, disrupts normal lipid metabolism, leading to the accumulation of triglycerides in hepatocytes, a condition known as NAFLD. This lipid overload further exacerbates oxidative stress and promotes inflammation, triggering hepatocyte apoptosis and fibrosis [9,10].

*Vinca rosea*, a medicinal plant traditionally used for its potential therapeutic benefits, has been reported to exhibit hypoglycemic, antioxidant, and anti-inflammatory properties [11,12]. These attributes make it a promising candidate for managing diabetic complications, particularly in mitigating liver damage [13,14]. However, while *Vinca rosea* has shown potential, scientific validation of its effects specifically on hepatic function in diabetic conditions remains limited.

This study aimed to evaluate the therapeutic effects of *Vinca rosea* leaf extract on hepatic function in STZ-induced diabetic Wistar albino rats. The primary objective was to assess the impact of *Vinca rosea* on blood glucose levels and key markers of hepatic function, including serum bilirubin, AST, and ALT alongside histopathological changes in liver tissue.

## Materials and methods

Study design and duration

This study was conducted at Roland Institute of Pharmaceutical Sciences, Berhampur from August 2023 to February 2024. To study the effects of *Vinca rosea* leaf extract on kidney and liver function, an experiment was set up using STZ-induced diabetic Wistar albino rats.

Animals

A total of 30 male Wistar albino rats weighing between 200 and 250 g were used in the study. The rats were housed in controlled environmental conditions, with a 12-hour light/dark cycle and free access to food and water. Prior to the commencement of the experiment, the rats were acclimatized for one week.

Induction of diabetes

Diabetes was induced in the rats by administering a single intraperitoneal injection of STZ at a dose of 55 mg/kg body weight, dissolved in a 100 mmol citrate buffer with an acidic pH of 4.0 to 4.5. To reduce mortality, nicotinamide at a dose of 120 mg/kg body weight was given 40 minutes prior to the STZ injection. STZ selectively damages insulin-producing beta cells in the pancreas, resulting in elevated blood glucose levels [7,8]. Blood glucose was measured 48 hours post-injection, and rats with fasting blood glucose levels of 180 mg/dL or higher were confirmed as diabetic and included in the study.

Grouping and treatment

The diabetic rats were randomly divided into five groups, with six rats in each group (n=6 per group), to ensure adequate statistical power and reliability of results. Group 1 served as the normal control (NC), consisting of non-diabetic rats receiving no treatment, representing baseline physiological conditions. Group 2, the diabetic control (DC), included untreated diabetic rats to observe the natural progression of diabetes and its complications. Group 3 (LD 200 mg/kg) comprised diabetic rats treated with a low dose of *Vinca rosea* leaf extract (200 mg/kg body weight), while Group 4 (HD 400 mg/kg) included diabetic rats treated with a high dose of *Vinca rosea* leaf extract (400 mg/kg body weight) to assess dose-dependent therapeutic effects. Group 5, the positive control (PC), consisted of diabetic rats treated with standard anti-diabetic therapy (like metformin), serving as a benchmark for evaluating the efficacy of *Vinca rosea*.

Including six rats per group allows for the detection of statistically significant differences between treatments while accounting for biological variability. This sample size strikes a balance between ethical considerations, cost, and the need to ensure the reproducibility and reliability of the experimental outcomes. Treatment with *Vinca rosea* leaf extract was administered orally once daily for 30 days, starting 48 hours after the confirmation of diabetes.

Sample collection and monitoring

Blood samples were collected from all rats via retro-orbital puncture under light ether anesthesia at two intervals: Day 15 and day 30. Blood glucose levels were measured using a glucometer. Serum was separated from the blood samples to evaluate hepatic function (serum bilirubin, AST and ALT).

Histopathological analysis

After the 30-day treatment period, liver tissue from each group was collected for histopathological analysis. The tissues were fixed in 10% formalin, then processed and embedded in paraffin. Thin sections of 5 microns were prepared and stained with hematoxylin and eosin (H&E) for microscopic evaluation. The sections were assessed for signs of hepatocyte degeneration, vacuolation, necrosis, and regeneration.

Biochemical analysis

Blood Glucose

Blood glucose levels were measured using a glucometer at baseline [12] on days 15 and 30.

Liver Function Tests (LFT)

Serum total bilirubin, direct bilirubin, AST, and ALT were measured to assess hepatic function [10].

Statistical analysis

The results are expressed as mean ± standard deviation (SD). Statistical analyses were performed using one-way analysis of variance (ANOVA) to evaluate differences among the experimental groups, followed by post hoc tests for pairwise comparisons to identify specific group differences. A p-value of less than 0.05 was considered statistically significant.

## Results

Blood glucose levels

Blood glucose levels were significantly elevated in the DC group compared to the NC group at both 15 and 30 days. At 15 days, the DC group exhibited blood glucose levels of 256 ± 19.8 mg/dL, while the NC group had levels of 93.94 ± 2.2 mg/dL. Treatment with *Vinca rosea* led to a dose-dependent reduction in blood glucose levels. The LD 200 mg/kg group showed glucose levels of 157 ± 9.6 mg/dL, while the HD 400 mg/kg group had glucose levels of 143 ± 12.7 mg/dL. The PC group, which received standard treatment, exhibited glucose levels of 133.2 ± 7.6 mg/dL (Table 1).

**Table 1:** Blood glucose levels in diabetic Wistar albino rats Low dose 200: Low-dose *Vinca rosea* group (200 mg/kg); High dose 400: High-dose *Vinca rosea* group (400 mg/kg) The DC group exhibited significantly elevated glucose levels compared to the normal control group. Treatment with *Vinca rosea* showed dose-dependent reductions in blood glucose, with the HD group demonstrating significant improvement, closely resembling the PC group. Statistical analysis using one-way analysis of variance (ANOVA) revealed highly significant differences among groups (p<0.0001), with pairwise comparisons confirming these findings.

Group	Blood Glucose - 15 Days (mg/dL)	Blood Glucose - 30 Days (mg/dL)
Normal Control (NC)	93.94 ± 2.2	93.94 ± 2.7
Diabetic Control (DC)	256 ± 19.8	308.5 ± 13.1
Low Dose 200	157 ± 9.6	181.8 ± 5.7
High Dose 400	143 ± 12.7	158.5 ± 10.7
Positive Control (PC)	133.2 ± 7.6	146.7 ± 5.8

At 30 days, the DC group exhibited a marked increase in blood glucose levels to 308.5 ± 13.1 mg/dL, while *Vinca rosea* treatment reduced glucose levels to 181.8 ± 5.7 mg/dL (LD 200 mg/kg) and 158.5 ± 10.7 mg/dL (HD 400 mg/kg). The PC group showed further improvement, with glucose levels of 146.7 ± 5.8 mg/dL (p < 0.001) (Table 1).

The statistical analysis of blood glucose levels revealed highly significant differences among the groups, as indicated by the extremely low p-values obtained from the one-way ANOVA. For the 15-day blood glucose levels, the ANOVA yielded a p-value of p=5.63×10^−92^, while for the 30-day levels, the p-value was p=2.33×10^−136^. These results confirm significant variations in blood glucose levels across the experimental groups.

Pairwise comparisons further substantiated these differences. At 15 days, the DC group exhibited significantly higher glucose levels compared to the NC group (p=1.12×10^−44^). Treatment with *Vinca rosea* led to dose-dependent reductions in blood glucose levels. The LD group differed significantly from the DC group (p=1.41×10^−30^) and the HD group (p=1.82×10^−5^). The HD group showed marked improvements compared to the DC group (p=1.45×10^−31^) and closely approached the glucose levels of the PC group (p=0.0001).

At 30 days, similar trends were observed. The DC group had significantly higher glucose levels compared to the NC group (p=2.71×10^−65^), while both the LD (p=1.32×10^−50^) and HD (p=1.33×10^−49^) groups showed significant reductions. The HD group demonstrated improved glucose control compared to the LD group (p=4.46×10^−16^) and was closer to the PC group (p=5.69×10^−5^).

These findings highlight the dose-dependent hypoglycemic effects of *Vinca rosea*, with the high-dose treatment achieving outcomes comparable to standard anti-diabetic therapy. This underscores its potential efficacy in managing hyperglycemia in diabetic conditions.

Liver function (serum bilirubin, AST, and ALT)

Total bilirubin levels were significantly elevated in the DC group at both 15 and 30 days. At 15 days, the DC group exhibited total bilirubin levels of 35.2 ± 2.4 mg/dL, while the NC group had levels of 16.4 ± 0.9 mg/dL. Treatment with *Vinca rosea* resulted in a reduction in total bilirubin levels, with the LD 200 mg/kg group showing levels of 25 ± 4 mg/dL and the HD 400 mg/kg group showing levels of 21 ± 5.6 mg/dL. After 30 days, the DC group exhibited total bilirubin levels of 38.7 ± 2.4 mg/dL, while *Vinca rosea* treatment reduced total bilirubin levels to 28 ± 4 mg/dL (LD) and 24 ± 5.6 mg/dL (HD) (Table 2).

**Table 2:** Liver function (serum bilirubin, AST, and ALT) in diabetic Wistar albino rats AST: aspartate aminotransferase; ALT: alanine aminotransferase; Low Dose 200: diabetic rats treated with a low dose of *Vinca rosea* leaf extract (200 mg/kg body weight); High Dose 400: diabetic rats treated with a high dose of *Vinca rosea* leaf extract (400 mg/kg body weight) Total bilirubin, direct bilirubin, AST, and ALT levels, at 15 and 30 days across experimental groups. The DC group exhibited significantly elevated levels compared to the NC, indicating severe hepatic dysfunction. Treatment with *Vinca rosea* resulted in dose-dependent improvements, with the high-dose group showing significant reductions and aligning closely with the positive control group. One-way analysis of variance (ANOVA) confirmed significant differences among groups (p<0.0001), supported by pairwise comparisons.

Group	Total Bilirubin: 15 Days (mg/dL)	Total Bilirubin: 30 Days (mg/dL)	Direct Bilirubin: 15 Days (mg/dL)	Direct Bilirubin: 30 Days (mg/dL)	AST: 15 Days (U/L)	AST: 30 Days (U/L)	ALT: 15 Days (U/L)	ALT: 30 Days (U/L)
Normal Control (NC)	16.4 ± 0.9	17.4 ± 0.9	3.5 ± 0.9	3.9 ± 0.9	15.7 ± 0.9	16.4 ± 0.9	35.2 ± 0.9	36.3 ± 0.9
Diabetic Control (DC)	35.2 ± 2.4	38.7 ± 2.4	6.4 ± 2.4	7.2 ± 2.4	26.8 ± 2.4	31 ± 2.4	90 ± 2.4	95.7 ± 2.4
Low Dose 200	25 ± 4	28 ± 4	5.3 ± 4	5.9 ± 4	20.4 ± 4	24 ± 4	62.7 ± 4	84.4 ± 4
High Dose 400	21 ± 5.6	24 ± 5.6	3.9 ± 5.6	4.3 ± 5.6	18.4 ± 5.6	21 ± 5.6	45 ± 5.6	67 ± 5.6
Positive Control (PC)	18 ± 6.3	20.7 ± 6.3	3.5 ± 6.3	3.8 ± 6.3	14.7 ± 6.3	17.7 ± 6.3	40 ± 6.3	41.4 ± 6.3

Direct bilirubin levels followed a similar trend. At 15 days, direct bilirubin levels in the DC group were 6.4 ± 2.4 mg/dL compared to 3.5 ± 0.9 mg/dL in the NC group. *Vinca rosea* treatment reduced these levels to 5.3 ± 4 mg/dL (LD) and 3.9 ± 5.6 mg/dL (HD). At 30 days, direct bilirubin levels in the DC group increased to 7.2 ± 2.4 mg/dL, while *Vinca rosea* treatment reduced them to 5.9 ± 4 mg/dL (LD) and 4.3 ± 5.6 mg/dL (HD) (Table 2).

Elevated levels of AST and ALT in the DC group were indicative of liver damage. At 15 days, AST levels in the DC group were 26.8 ± 2.4 U/L compared to 15.7 ± 0.9 U/L in the NC group. *Vinca rosea* treatment reduced AST levels to 20.4 ± 4 U/L (LD) and 18.4 ± 5.6 U/L (HD). At 30 days, AST levels in the DC group increased to 31 ± 2.4 U/L, while *Vinca rosea* treatment reduced them to 24 ± 4 U/L (LD) and 21 ± 5.6 U/L (HD).

ALT levels followed a similar pattern. At 15 days, ALT levels in the DC group were 90 ± 2.4 U/L compared to 35.2 ± 0.9 U/L in the NC group. Treatment with *Vinca rosea* reduced ALT levels to 62.7 ± 4 U/L (LD) and 45 ± 5.6 U/L (HD). At 30 days, ALT levels in the DC group increased to 95.7 ± 2.4 U/L, while *Vinca rosea* treatment reduced them to 84.4 ± 4 U/L (LD) and 67 ± 5.6 U/L (HD) (Table 2).

The one-way ANOVA revealed highly significant differences among the experimental groups across all measured biochemical markers, including total bilirubin, direct bilirubin, AST, and ALT levels, at both 15 and 30 days.

Total Bilirubin

At 15 days, the ANOVA yielded a p-value of p=1.69×10^−32^, and at 30 days, p=2.29×10^−45^, indicating substantial group differences. Pairwise comparisons showed that the DC group exhibited significantly higher bilirubin levels compared to the normal NC group (e.g., p=1.86×10^−40^ at 15 days). *Vinca rosea* treatment (LD and HD groups) showed dose-dependent reductions, with the HD significantly different from the DC group (p=4.75×10^−14^) and closer to the PC group (p=0.03) at 15 days.

Direct Bilirubin

The ANOVA showed significant differences at 15 days (p=3.16×10^−5^) and 30 days (p=1.24×10^−6^). Pairwise analysis revealed that the DC group had markedly elevated levels compared to the NC group (p=4.25×10^−8^), while *Vinca rosea*-treated groups exhibited improvements, particularly in the HD group, which showed significant differences from the DC group (p=1.34×10^−4^).

AST Levels

Significant differences were observed at 15 days (p=1.22×10^−20^) and 30 days (p=1.03×10^−31^). The DC group showed elevated AST levels compared to the NC group (p=1.27×10^−28^ at 15 days), while the HD group demonstrated reductions, differing significantly from the DC group (p=9.26×10^−11^) and approaching levels seen in the PC group.

ALT Levels

ALT levels were highly elevated in the DC group compared to the NC group (p=6.15×10^−74^ at 15 days, p=5.99×10^−78^ at 30 days). *Vinca rosea* treatment led to dose-dependent reductions in ALT, with the HD group showing significant differences from the DC group (p=3.42×10^−40^) and aligning closely with the PC group (p=0.008).

Histopathological analysis of the liver

In this study, the liver tissue across different treatment groups showed distinct histopathological changes, summarized as follows:

NC

**Figure 1:**
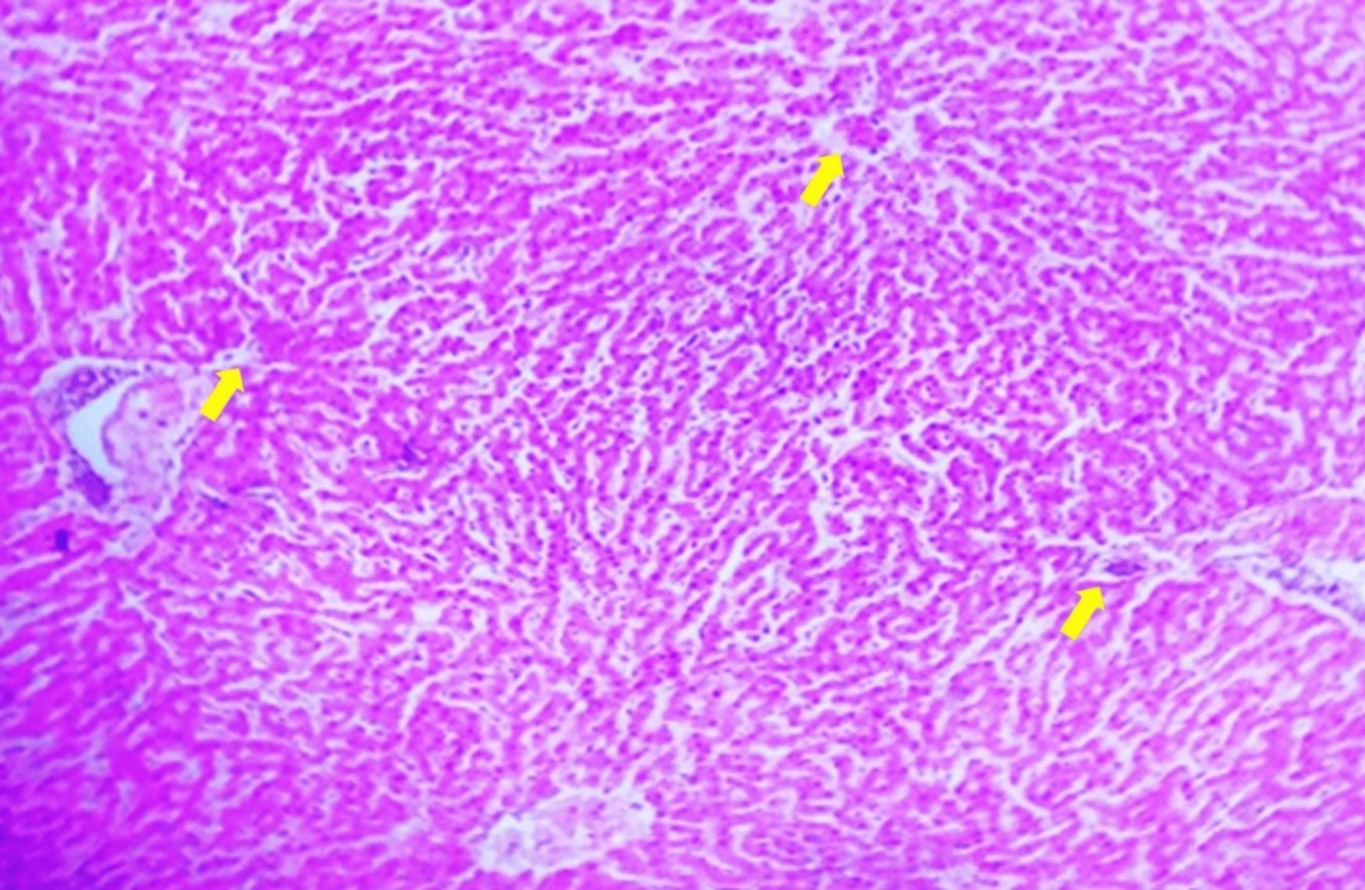
Normal control rat liver showing central vein with radiating hepatic cords. H&E staining of the tissue section, viewed under 10x magnification. Yellow arrows indicate areas of interest, highlighting specific morphological features within the tissue.

Rat liver tissue shows a clear central vein with radiating hepatic cords, displaying typical healthy liver morphology without any pathological changes (Figure 1).

DC

**Figure 2:**
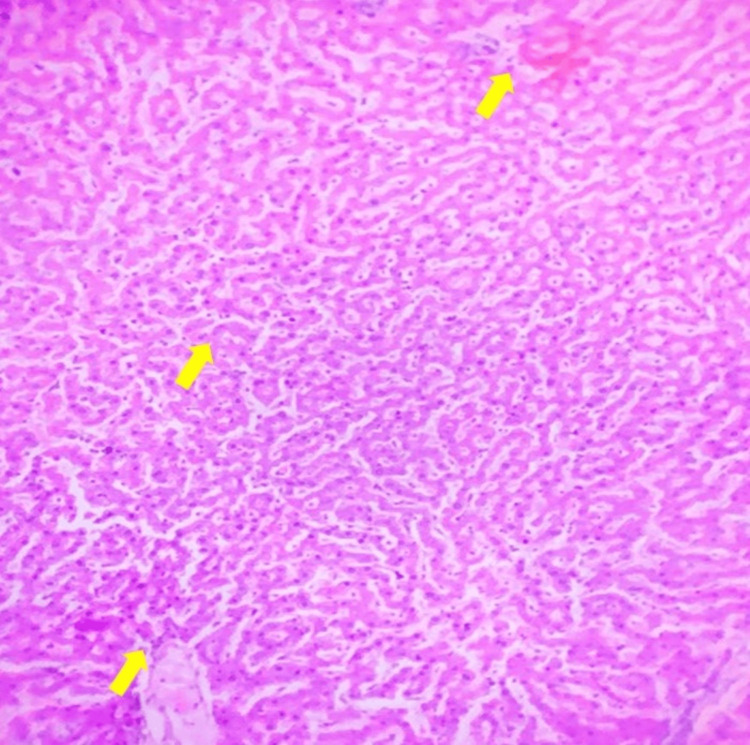
Diabetic control: After administration of streptozotocin (30 days), liver showing degenerative changes and necrosis with mild periportal infiltration. Tissue section stained with H&E, observed at 10x magnification.

After 30 days of STZ administration, liver tissue displays significant hepatocellular damage, including degeneration, necrosis, and mild perivascular infiltration, indicating the impact of diabetes on liver structure (Figure 2).

Low-Dose 200

**Figure 3:**
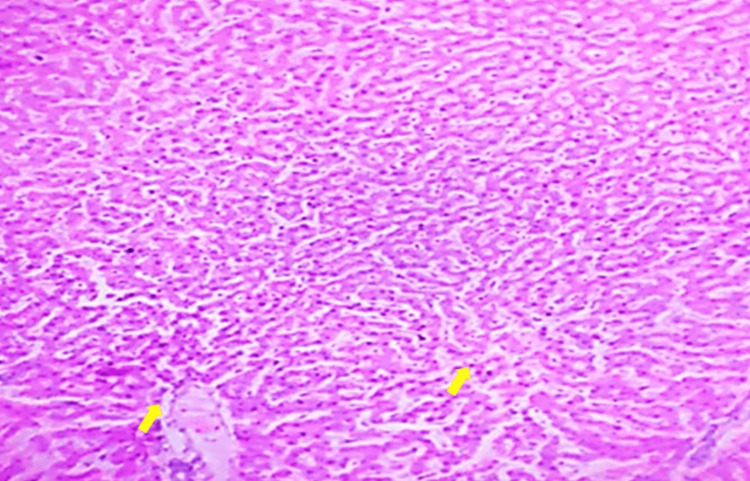
Liver tissue from a low-dose (200 mg/kg) Vinca rosea-treated group, showing slight resemblance to normal liver morphology. Tissue section stained with H&E, observed at 10x magnification.

Liver tissue from this group shows moderate improvements in histology compared to the diabetic control, with reduced vacuolation and indications of early hepatocyte regeneration, resembling normal liver morphology to some extent (Figure 3).

High-Dose 400

**Figure 4:**
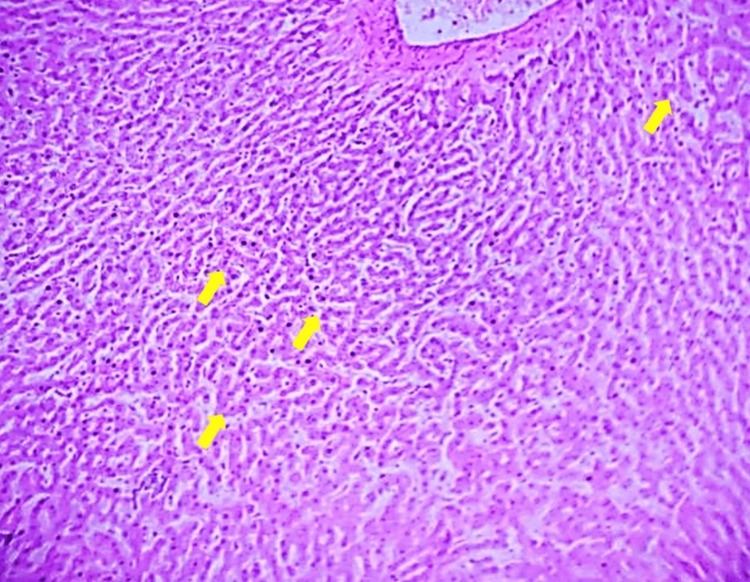
Liver tissue from a high-dose (400 mg/kg) Vinca rosea-treated group after 30 days of administration, showing notable resemblance to normal liver morphology. Tissue section stained with H&E, observed at 10x magnification.

After 30 days of administration, liver tissue exhibits a marked resemblance to normal morphology, with nearly restored liver architecture and minimal signs of damage, suggesting strong hepatoprotective effects at a higher dosage (Figure 4).

Metformin-Treated Group (100 mg/kg)

**Figure 5:**
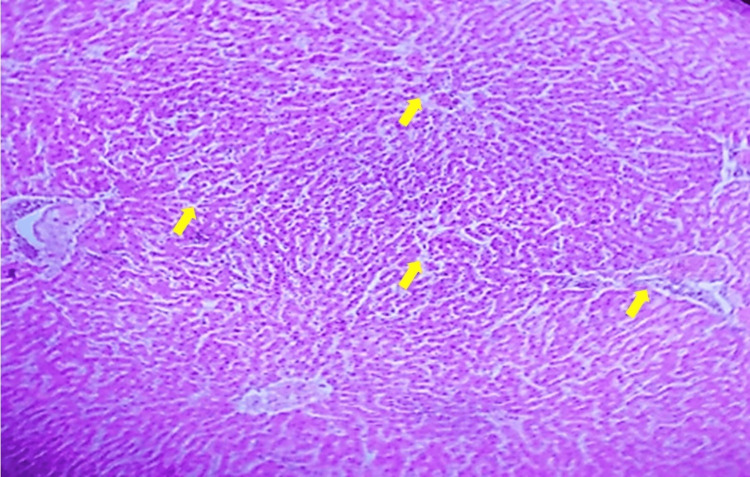
Liver tissue from the metformin-treated group (100 mg/kg) after 30 days of administration, showing resemblance to normal liver morphology. H&E staining of tissue section, viewed under 10x magnification.

Liver tissue from the metformin-treated group, after 30 days, shows significant recovery with morphology similar to the normal control, indicating minimal pathological changes and the therapeutic efficacy of metformin in restoring hepatic health (Figure 5).

Each group was analyzed to assess the hepatoprotective effects of *Vinca rosea* in both low and high doses, compared with the positive control metformin group and the normal control group. The diabetic control group (Figure 3) highlighted the extent of diabetic damage in untreated rats, while the high-dose *Vinca rosea* and metformin groups exhibited substantial recovery, approaching the structural normalcy seen in the normal control (Figure 2).

## Discussion

The findings from this study align with previous research that demonstrates the hepatoprotective and antidiabetic potential of plant-based therapies in diabetic models [6-8,12]. The hypoglycemic effects observed in *Vinca rosea*-treated groups, particularly in the high-dose (400 mg/kg) group, are consistent with prior studies indicating the role of natural compounds in managing blood glucose levels. Boro et al. (2022) highlighted similar protective effects in the ethanolic extract of *Morus indica* roots, which effectively reduced hyperglycemia and associated hepatic stress in STZ-induced diabetic models [15]. Hyperglycemia is a key factor leading to hepatic complications in diabetes, and the ability of *Vinca rosea* to lower blood glucose suggests it may improve glucose metabolism, enhance insulin sensitivity, or reduce oxidative stress, which has been a focal point in recent antidiabetic research from Singh et al. (2013) [16], Gadewar et al. (2023) [17]. *Vinca rosea* blood glucose levels align with findings from polyherbal preparations studied by Jayaraju et al. (2022), which similarly demonstrated blood glucose regulation and reduced liver stress [18].

Furthermore, the improved function markers (bilirubin, AST, ALT) following *Vinca rosea* treatment provide evidence of its hepatoprotective effects, similar to findings by Gupta et al. (2019), who reported that phytol-based treatments significantly lowered these markers in diabetic rat models [19]. Elevated levels of these enzymes in diabetic models typically signify liver damage due to oxidative stress and inflammation in hepatocytes [20]. Treatment with *Vinca rosea* notably reduced these markers, which supports the hypothesis of its role in preventing or reversing hepatic injury.

The histopathological analysis provided additional evidence supporting the therapeutic efficacy of *Vinca rosea,* revealing significant improvements in liver tissue structure in treated groups. The HD group, in particular, displayed liver architecture that closely resembled normal tissue, with minimal pathological changes. This suggests a potential regenerative or reparative effect of V*inca rosea* on hepatic tissues, possibly mediated through its antioxidant, anti-inflammatory, or cytoprotective properties. These findings align with previous studies on similar botanical interventions, where plant-derived natural compounds were shown to exert hepatoprotective effects by promoting cellular recovery, reducing oxidative stress, and minimizing histological and biochemical markers of liver damage. Such outcomes underscore the potential of *Vinca rosea* as a promising candidate for mitigating liver injury associated with diabetic conditions [21,22].

The therapeutic effects of *Vinca rosea* are likely attributed to its bioactive compounds, such as flavonoids and alkaloids, recognized for their antioxidant and anti-inflammatory properties. These compounds may help alleviate oxidative stress and inflammation in liver tissue, common complications in diabetes that often result in hepatic dysfunction. This mechanism supports findings by Boro et al. (2022) [15] and Singh et al. (2013) [16], who demonstrated that plant extracts rich in flavonoids provide substantial protection against oxidative damage in diabetic models.

Study limitations

Despite the promising findings, several limitations must be acknowledged. First, the study was conducted using an animal model, and further research is needed to confirm these effects in human clinical trials. Second, the study did not isolate and identify the specific bioactive compounds responsible for *Vinca rosea* therapeutic effects, warranting further phytochemical analysis. Lastly, the study only covered a 30-day treatment period. Long-term studies are necessary to evaluate the sustained effects of *Vinca rosea* on hepatic function and the safety of prolonged use.

## Conclusions

This study demonstrated that *Vinca rosea* leaf extract has significant hepatoprotective effects in STZ-induced diabetic Wistar albino rats. *Vinca rosea* treatment led to dose-dependent reductions in blood glucose levels and improvements in liver function, as evidenced by decreased serum bilirubin, AST, and ALT levels. Histopathological analysis further supported these findings, showing that *Vinca rosea*, particularly at the high dose (400 mg/kg), restored near-normal liver architecture and reduced hepatocellular damage.

The HD of *Vinca rosea* demonstrated the most significant therapeutic benefits, indicating its potential as an effective adjunct therapy for addressing diabetes-induced hepatic damage. Its notable ability to alleviate oxidative stress and inflammation, coupled with its apparent capacity to promote liver tissue regeneration, underscores its promise as a hepatoprotective agent. These findings highlight *Vinca rosea*'s potential role in mitigating hepatic complications associated with diabetes. Nevertheless, further investigations are warranted to identify and isolate the bioactive compounds responsible for these effects, elucidate the underlying molecular mechanisms, and evaluate their long-term safety and efficacy in preclinical and clinical settings. Such research will be crucial for translating its therapeutic potential into practical medical applications.

## References

[1] Antar SA, Ashour NA, Sharaky M (2023). Diabetes mellitus: classification, mediators, and complications; a gate to identify potential targets for the development of new effective treatments. Biomed Pharmacother.

[2] Hossain MJ, Al-Mamun M, Islam MR (2024). Diabetes mellitus, the fastest growing global public health concern: early detection should be focused. Health Sci Rep.

[3] Eid S, Sas KM, Abcouwer SF, Feldman EL, Gardner TW, Pennathur S, Fort PE (2019). New insights into the mechanisms of diabetic complications: role of lipids and lipid metabolism. Diabetologia.

[4] García-Compeán D, Orsi E, Kumar R (2022). Clinical implications of diabetes in chronic liver disease: diagnosis, outcomes and management, current and future perspectives. World J Gastroenterol.

[5] Yazdi HB, Hojati V, Shiravi A, Hosseinian S, Vaezi G, Hadjzadeh MA (2019). Liver dysfunction and oxidative stress in streptozotocin-induced diabetic rats: protective role of Artemisia turanica. J Pharmacopuncture.

[6] Saeed MK, Deng Y, Dai R (2008). Attenuation of biochemical parameters in streptozotocin-induced diabetic rats by oral administration of extracts and fractions of Cephalotaxus sinensis. J Clin Biochem Nutr.

[7] Alqahtani QH, Alshehri S, Alhusaini AM (2023). Protective effects of sitagliptin on streptozotocin-induced hepatic injury in diabetic rats: a possible mechanisms. Diseases.

[8] Bakaç MS, Dogan A, Yılmaz MA, Altındag F, Donmez F, Battal A (2023). Ameliorative effects of Scutellaria Pinnatifida subsp. pichleri (Stapf) Rech.f. Extract in streptozotocin-induced diabetic rats: chemical composition, biochemical and histopathological evaluation. BMC Complement Med Ther.

[9] Teshome G, Ambachew S, Fasil A, Abebe M (2019). Prevalence of liver function test abnormality and associated factors in type 2 diabetes mellitus: a comparative cross-sectional study. EJIFCC.

[10] Zheng D, Zhang X, You L (2022). The association of liver enzymes with diabetes mellitus risk in different obesity subgroups: a population-based study. Front Endocrinol (Lausanne).

[11] Verma R (2018). A review on hepatoprotective activity of medicinal plants. J Med Plants Stud.

[12] Ahmed MF, Kazim SM, Ghori SS, Mehjabeen SS, Ahmed SR, Ali SM, Ibrahim M (2010). Antidiabetic activity of Vinca rosea extracts in alloxan-induced diabetic rats. Int J Endocrinol.

[13] Ghosh S, Suryawanshi SA (2001). Effect of Vinca rosea extracts in treatment of alloxan diabetes in male albino rats. Indian J Exp Biol.

[14] Avanapu SR, Ahmad D (2010). Phytochemical evaluation and hepatoprotective activity of Catharanthus rosea against simvastatin-induced hepatotoxicity in rats. Int J Adv Pharm Med Bioallied Sci.

[15] Boro H, Usha T, Babu D (2022). Hepatoprotective activity of the ethanolic extract of Morus indica roots from Indian Bodo tribes. SN Appl Sci.

[16] Singh R, Bhardwaj P, Sharma P (2013). Antioxidant and toxicological evaluation of Cassia sopherain streptozotocin-induced diabetic Wistar rats. Pharmacognosy Res.

[17] Gadewar MM, G K P, Mishra PC (2023). Evaluation of antidiabetic, antioxidant and anti-hyperlipidemic effects of Solanum indicum fruit extract in streptozotocin-induced diabetic rats. Curr Issues Mol Biol.

[18] Jayaraju KJ, Ishaq BM (2022). Antidiabetic and hepatoprotective activity of a novel polyherbal preparation against streptozotocin-induced diabetes rats and its formulation into a tablet dosage form. Asian J Pharm Res Health Care.

[19] Gupta K, Taj T, Thansiya B, Kamath JV (2019). Pre-clinical evaluation of hepatoprotective activity of phytol in wistar albino rats. Int J Compr Adv Pharmacol.

[20] El-Demerdash FM, Talaat Y, El-Sayed RA, Kang W, Ghanem NF (2022). Hepatoprotective effect of Actinidia deliciosa against streptozotocin-induced oxidative stress, apoptosis, and inflammations in rats. Oxid Med Cell Longev.

[21] Chitra P, Hemalakshmi M (2020). Anti-diabetic and anti-hyperlipidemic effects of polyherbal ethanolic extracts(allium sativum, Vinca rosea & Mangifera indica) in streptozotocin induced hyperglycemic rats. Eur J Mol Clin Med.

[22] Reddy NS, Vidyasabbani B, Pravanthi B, Vijaya Laxmi B, Harika Harika (2016). Evaluation of antidiabetic activity of Rumex vesicarius in streptozotocin induced diabetic albino rats. Res J Pharmacology & Pharmacodynamics.

